# Thresholds for optimal fluid administration and weight gain after laparoscopic colorectal surgery

**DOI:** 10.1002/bjs5.50166

**Published:** 2019-04-02

**Authors:** M. Hübner, B. Pache, J. Solà, C. Blanc, D. Hahnloser, N. Demartines, F. Grass

**Affiliations:** ^1^ Department of Visceral Surgery Lausanne University Hospital, Centre Hospitalier Universitaire Vaudois Lausanne Switzerland; ^2^ Department of Anaesthesiology Lausanne University Hospital, Centre Hospitalier Universitaire Vaudois Lausanne Switzerland; ^3^ Centre Suisse d'Electronique et de Microtechnique Neuchâtel Switzerland

## Abstract

**Background:**

Perioperative fluid overload is an important modifiable risk factor for adverse outcomes after colorectal surgery. This study aimed to define critical thresholds for perioperative fluid management and postoperative weight gain for patients undergoing elective laparoscopic colorectal surgery.

**Methods:**

This was an analysis of consecutive elective laparoscopic colorectal resections at Lausanne University Hospital from May 2011 to May 2017. Main outcomes were overall, major (Clavien–Dindo grade IIIb or above) and respiratory complications, and postoperative ileus. Thresholds regarding perioperative fluid management and postoperative weight gain were identified through receiver operating characteristic (ROC) analysis and clinical judgement. Independent risk factors for all four outcomes were assessed by multinominal logistic regression.

**Results:**

Overall and major complications occurred in 210 (36·2 per cent) and 46 (7·9 per cent) of 580 patients respectively. Twenty‐three patients (4·0 per cent) had respiratory complications and 98 (16·9 per cent) had postoperative ileus. Median length of hospital stay was 5 (i.q.r. 3–9) days. Based on respiratory complications, thresholds for perioperative intravenous fluid administration (postoperative day (POD) 0) were set pragmatically at 3000 ml for colonic (calculated threshold 3120 ml (area under ROC curve (AUROC) 0·63)) and 4000 ml for rectal (AUROC 0·79) procedures. Postoperative weight gain of 2·5 kg at POD 2 was predictive of respiratory complications. Multivariable analysis retained perioperative intravenous fluid administration over the above thresholds as an independent risk factor for overall (odds ratio (OR) 2·25, 95 per cent c.i. 1·23 to 4·11), major (OR 2·49, 1·17 to 5·31) and respiratory (OR 4·71, 1·42 to 15·58) complications. Weight gain above 2·5 kg at POD 2 was identified as a risk factor for respiratory complications (OR 3·58, 1·10 to 11·70) and ileus (OR 1·82, 1·02 to 3·52).

**Conclusion:**

Perioperative intravenous fluid and weight thresholds were associated with postoperative adverse outcomes. These thresholds need independent validation.

## Introduction

Stringent perioperative fluid management is a key component of enhanced recovery after surgery (ERAS) programmes, challenging traditional care schemes in many ways^1^, ^2^, ^3^. The patient is allowed to drink clear liquids and carbohydrate drinks until 2 h before surgery^4^, a zero‐fluid balance is aimed for during the surgical procedure^5^ and high‐risk patients are managed according to the principle of goal‐directed fluid therapy, which was most beneficial in traditional care pathways^6^, ^7^, ^8^. Decreased urine output is no longer considered harmful and should not be the primary guide to fluid management^5^, ^9^. After surgery, early weaning of intravenous fluids and transition to oral fluids complete this perioperative strategy, which has repeatedly been associated with decreased postoperative morbidity^10^, ^11^. Within an enhanced recovery concept, minimally invasive surgery adds further advantages via a synergistic beneficial effect^12^, ^13^, ^14^. To date, recommendations for perioperative fluid administration remain arbitrary, and boundary values have not been defined.

The present study aimed to identify critical thresholds regarding perioperative intravenous fluid management and postoperative weight gain after elective laparoscopic colonic resections to facilitate guidance in daily clinical practice.

## Methods

Consecutive patients undergoing elective laparoscopic colorectal resections over a 6‐year period (May 2011 to May 2017) at Lausanne University Hospital were included. Patients were treated within a standard ERAS pathway over the study interval^15^. Data were entered into an institutional ERAS database by an institutional ERAS nurse, and cross‐checked during weekly audit sessions by the institutional ERAS team (internal validation). Items in this database have been described previously^16^, ^17^. All colorectal resections were standardized and performed by the institutional colorectal team, which included three senior staff surgeons. Procedures were assigned as either colonic or rectal laparoscopic resections, comprising low anterior resections, proctocolectomies or abdominoperineal resections. All emergency procedures were excluded. Converted procedures were not excluded according to the intention‐to‐treat principle.

### Ethical considerations

This study was considered an institutional quality improvement project; data extraction was approved by the Institutional Review Board (Commission Cantonale d'Ethique de la Recherche sur l'être humain CER‐VD number 2017‐01991). The study was conducted in line with the declaration of Helsinki and STROBE criteria^18^.

### Assessment of perioperative fluid management and postoperative weight

Three parameters were assessed: total intravenous administration on the day of surgery (postoperative day (POD) 0), comprising liquids administered during surgery (crystalloids, colloids and blood products) and those administered after surgery until midnight of POD 0 (perioperative fluids), using data from chart review of anaesthesia protocols; the amount of intraoperative intravenous fluid administered (weight and duration‐adjusted volume: ml per kg per h), where balanced administration was defined^19^ as less than 7 ml per kg per h; and postoperative weight, assessed on POD 1–3 by the staff nurse on the ward using standard balances. All three fluid‐related parameters were analysed to identify relevant cut‐offs for postoperative adverse outcomes, as defined below. Subgroup analysis was performed for rectal procedures.

### Outcomes/study endpoints

Endpoints were overall complication rate (Clavien–Dindo grade I–V)^20^, major complications (Clavien–Dindo grade IIIb or above), respiratory complications and postoperative ileus. Respiratory complications were defined as radiologically confirmed pneumonia requiring antibiotic treatment, lobar atelectasis needing physiotherapy beyond standard use of incentive spirometry six times daily, pleural effusion necessitating surgical or radiologically guided drainage, and respiratory failure requiring transfer to an intermediate or intensive care unit^21^. Postoperative ileus was defined as time to stool beyond POD 3^22^. All endpoints were assessed to POD 30 (in‐hospital and at outpatient visits).

Length of hospital stay and overall compliance with the ERAS protocol greater than 70 per cent in patients with complete data sets for all 22 preoperative, perioperative and postoperative ERAS items^23^ were also measured.

### Statistical analysis and assessment of thresholds

Optimal thresholds for each fluid‐related parameter (perioperative and intraoperative fluids, and those on POD 1–3) were assessed by receiver operating characteristic (ROC) curve analysis. ROC curves were calculated with the Statistical and Machine Learning Toolbox™ of MATLAB R2018a (Mathworks, Natick, Massachusetts, USA). Confidence intervals for the area under the curve (AUC) were calculated by bootstrapping on 1000 replicas. Optimal operating points were determined mathematically as those points that jointly optimized the sensitivity and specificity of each ROC curve. Finally, optimal clinical thresholds were defined considering the decisional criteria of high negative predictive potential, early diagnosis, specificity and practicability.

Descriptive statistics for categorical variables are reported as frequencies, and continuous variables as mean(s.d.) or median (i.q.r.) values. The χ^2^ test was used for comparison of categorical variables, whereas Student's *t* test was used to compare continuous variables. All statistical tests were two‐sided, and a level of 0·050 was used to indicate statistical significance.

Fluid thresholds, together with demographic and surgical risk factors that were significant in univariable analysis, were included into a multinominal logistic regression model to calculate adjusted odds ratios (ORs) for the four main outcomes (overall, major and respiratory complications, and postoperative ileus).

Data analysis was performed with SPSS® Advanced Statistics 22 (IBM, Armonk, New York, USA).

## Results

### Patients and outcomes

The final analysis included 580 patients (*Table* 1). The conversion rate to open surgery was 8·3 per cent (48 procedures). These patients were included for further analysis according to the intention‐to‐treat principle. Overall complications were observed in 210 patients (36·2 per cent) and major complications in 46 (7·9 per cent). Twenty‐three patients (4·0 per cent) developed respiratory complications and 98 (16·9 per cent) had postoperative ileus. Median length of stay was 5 (i.q.r. 3–9) days. In univariable analysis patients with complications had a longer duration of surgery and more perioperative fluid administration and postoperative weight gain than patients without complications (*Table* 2).

**Table 1:** Baseline demographics of patients with and without complications

	All patients (*n* = 580)	No complication (*n* = 370)	Any complication (*n* = 210)	*P* †
Age (years)*	62(16)	61(16)	62(17)	0·746‡
Age ≥ 70 years	212 (36·6)	130 (35·1)	82 (39·0)	0·370
Sex ratio (M : F)	314 : 266	197 : 173	117 : 93	0·603
BMI (kg/m^2^)*	25·9(5·1)	25·6(5·1)	26·1(5·1)	0·285‡
ASA fitness grade				0·139
I–II	456 (78·8)	299 (80·8)	158 (75·2)	
III–IV	124 (21·4)	71 (19·2)	52 (24·8)	
Smoker	123 (21·2)	74 (20·0)	49 (23·3)	0·344
Previous abdominal surgery	154 (26·6)	89 (24·1)	65 (31·0)	0·071
Malignancy	413 (71·2)	260 (70·3)	153 (72·9)	0·412

Values in parentheses are percentages unless indicated otherwise;

* values are mean(s.d.).

† χ^2^ test, except

‡ Student's *t* test.

**Table 2:** Surgical and enhanced recovery after surgery‐related parameters

	All patients (*n* = 580)	No complication (*n* = 370)	Any complication (*n* = 210)	*P* ‡
Duration of surgery (min)*	210(90)	190(70)	230(100)	< 0·001§
Duration of surgery > 180 min	286 (49·3)	157 (42·4)	129 (61·4)	< 0·001
Administration of i.v. fluid				
Total during surgery (ml)*	1900(1000)	1700(700)	2200(1200)	< 0·001§
Total at POD 0 (ml)*	2800(1300)	2500(1000)	3200(1500)	< 0·001§
Total at POD 0 above threshold†	122 (21·0)	53 (14·3)	69 (32·9)	< 0·001
Volume during surgery > 7 ml per kg per h	314 (54·1)	193 (52·2)	121 (57·6)	0·204
Rectal surgery	191 (32·9)	101 (27·3)	90 (42·9)	0·001
Compliance with ERAS > 70%	289 of 442 (65·4)	213 of 285 (74·7)	76 of 157 (48·4)	< 0·001
Weight gain at POD 2 (kg)*	1·3(2·5)	1·0(2·2)	1·9(2·9)	0·002
Weight gain > 2·5 kg at POD 2	110 of 413 (26·6)	57 of 264 (21·6)	53 of 149 (35·6)	0·003

Values in parentheses are percentages unless indicated otherwise;

* values are mean(s.d.).

† Threshold 3 litres for colonic and 4 litres for rectal resections. POD, postoperative day; i.v., intravenous; ERAS, enhanced recovery after surgery.

‡ χ^2^ test, except

§ Student's *t* test.

### Thresholds for fluid administration and postoperative weight gain

**Figure 1:**
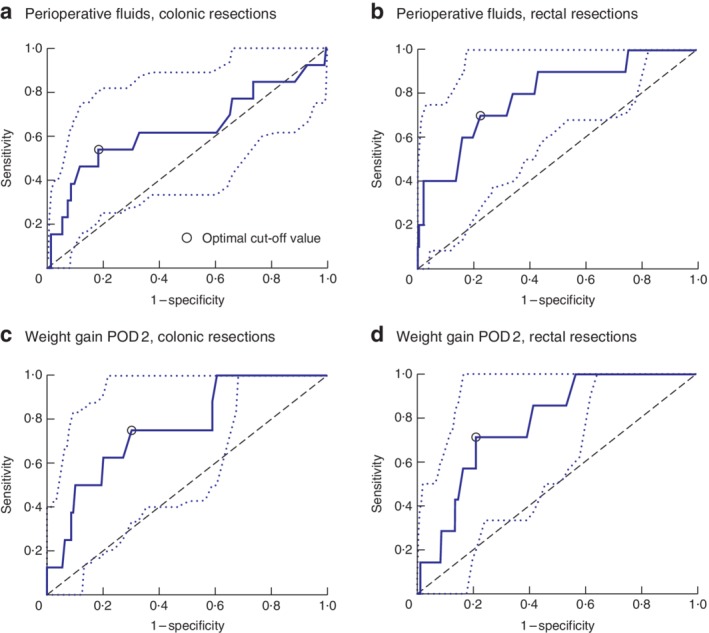
Receiver operating characteristic (ROC) curves. ROC curves for **a,b** total intravenous fluid administration at postoperative day (POD) 0 (perioperative fluids) and **c,d** weight gain at POD 2 and respiratory complications (23 patients). **a,c** Colonic and **b,d** rectal resections. **a** Area under the curve (AUC) 0·63 (95 per cent c.i. 0·43 to 0·84), threshold 3120 ml; **b** AUC 0·79 (0·62 to 0·93), threshold 4000 ml; **c** AUC 0·76 (0·58 to 0·92), threshold 2·3 kg; **d** AUC 0·78 (0·6 to 0·91), threshold 2·7 kg

Thresholds (optimal operating point in the AUC) for intraoperative fluid administration, perioperative fluids and postoperative weight gain on POD 1, 2 and 3 were calculated for the four outcomes of interest (overall, major and respiratory complications, and ileus) (*Table* 3). Intraoperative fluid administration had a low predictive potential throughout. Respiratory complications appeared to be the most specific outcome related to fluid administration and weight gain (*Table* 3). Based on these findings and the decisional criteria, perioperative fluids and weight gain on POD 2 were identified as predictive parameters. Perioperative fluid thresholds differed for laparoscopic colonic and rectal resections: 3000 and 4000 ml respectively (*Fig*. 1). Small differences were observed between the two types of surgery (colonic or rectal) for postoperative weight gain (2·7 and 2·3 kg respectively), so the threshold on POD 2 was set at 2·5 kg.

**Table 3:** Receiver operating characteristic (ROC) curve analysis

	Intraoperative fluids (ml per kg per h)	Perioperative fluids (ml)	Postoperative weight gain (kg)		
			POD 1	POD 2	POD 3
Complications					
Overall	7·4 (0·54)	2650 (0·65)	1·2 (0·54)	1·1 (0·61)	0·5 (0·63)
Major	7·4 (0·53)	3100 (0·65)	2·4 (0·61)	1·4 (0·62)	1·7 (0·61)
Respiratory	7·6 (0·51)	3120 (0·63)	2·5 (0·57)	2·3 (0·76)	1·5 (0·79)
Ileus	7·4 (0·53)	2950 (0·55)	1·2 (0·56)	1·4 (0·64)	1·4 (0·64)

Values are optimal mathematical thresholds with associated area under the curve (AUC) in parentheses. POD, postoperative day.

### Perioperative fluid administration, POD 2 weight gain and complications

Sensitivity, specificity, positive (PPV) and negative predictive (NPV) values are shown in *Table* S1 (supporting information). NPVs were 69, 94 and 98 per cent for overall, severe and respiratory complications respectively.

Perioperative fluid administration and POD 2 weight gain above the defined thresholds were entered, together with demographic and surgical risk factors, into a multinominal logistic regression model (*Table* 4). Intravenous fluid administration above the threshold at POD 0 was independently associated with overall, major and respiratory complications. POD 2 weight gain above the threshold was independently associated with respiratory complications and postoperative ileus.

**Table 4:** Multivariable analysis

	Any complication		Major complication		Respiratory complication		Ileus	
	OR	*P*	OR	*P*	OR	*P*	OR	*P*
ASA grade ≥ III (*versus* grade I–II)	–		–		2·31 (0·74, 7·25)	0·151	2·88 (1·51, 5·49)	0·001
Smoker (*versus* non‐smoker)	–		–		2·24 (0·69, 7·26)	0·181	–	
Duration of surgery > 180 min (*versus* ≤ 180 min)	1·41 (0·78, 2·55)	0·263	2·51 (0·9, 7·02)	0·080	–		–	
Rectal surgery (*versus* colonic surgery)	1·53 (0·86, 2·71)	0·147	1·93 (0·9, 4·18)	0·094	–		–	
Compliance with ERAS > 70% (*versus* ≤ 70%)	0·53 (0·32, 0·88)	0·015	0·55 (0·27, 1·15)	0·113	–		0·73 (0·39, 1·37)	0·327
Weight gain at POD 2 > 2·5 kg (*versus* ≤ 2·5 kg)	1·27 (0·73, 2·22)	0·403	–		3·58 (1·10, 11·70)	0·034	1·82 (1·02, 3·52)	0·049
i.v. fluids above threshold on POD 0 (*versus* below threshold)*	2·25 (1·23, 4·11)	0·008	2·49 (1·17, 5·31)	0·018	4·71 (1·43, 15·58)	0·011	0·9 (0·43, 1·91)	0·786

Values in parentheses are 95 per cent confidence intervals.

* Threshold 3 litres for colonic and 4 litres for rectal resections. OR, odds ratio; ERAS, enhanced recovery after surgery; POD, postoperative day; i.v., intravenous.

## Discussion

Perioperative fluid administration greater than 3 litres for colonic and 4 litres for rectal procedures, and weight gain of more than 2·5 kg on POD 2 were associated with adverse outcomes after elective laparoscopic colorectal surgery. These thresholds may be used in future work to investigate optimal fluid administration.

Perioperative fluid management is a key item of enhanced recovery protocols and part of the overall strategy designed to decrease the surgical stress response^1^, ^4^. An RCT^24^ of fluid restriction compared with oesophageal Doppler‐guided goal‐directed fluid therapy in elective colorectal surgery within an ERAS programme did not show any advantage of goal‐directed therapy in terms of length of stay or morbidity. A recent large‐scale randomized trial^25^ failed to demonstrate increased disability‐free survival in patients receiving a restrictive fluid regimen compared with that in patients receiving a liberal fluid regimen after major abdominal surgery. In fact, a higher rate of acute kidney injury (8·6 *versus* 5 per cent) was observed in the restrictive fluid group, although 50 per cent of patients were not treated within an enhanced recovery pathway and fluid restriction did not increase the risk of acute kidney injury in patients treated within an ERAS pathway. A recent large‐scale study^26^ analysed fluid administration practices across 64 hospitals, and found wide variation with a correlation between high fluid balances and prolonged, risk‐adjusted, length of stay.

In the present study three parameters were explored to define critical thresholds. Weight and duration‐adjusted volume (ml per kg per h) was not a reliable predictor of postoperative complications in the present study and has not been retained in recent guidelines^5^. Measurement of the total amount of intravenous fluid administered by the end of the day of surgery indicated a mean fluid load of 2·8 litres, consistent with reported ranges in similar settings^27^, ^28^, ^29^. The present study identified a threshold for adverse outcomes of 3 litres for colonic and 4 litres for rectal procedures. Weight gain was assessed during the first 3 days after surgery and considered useful in the elective setting, as it is measured easily. A POD 2 weight gain of 2·5 kg was retained as a pragmatic critical threshold, as subsequent treatments for patients exceeding this limit can be launched in a timely way.

ROC curve analysis revealed the thresholds to be most significant for respiratory complications, which occurred in 4·0 per cent of the study population, similar to findings in other studies of laparoscopic surgery^30^. These thresholds were found to be independent predictors of further adverse events. Of note, in this study postoperative weight gain greater than 2·5 kg was associated with an approximately twofold increased risk of postoperative ileus, similar to other reports^31^, ^32^.

From the clinicians' perspective, thresholds should be pragmatic, easily assessable and, ideally, highly predictive. Although a threshold for total perioperative fluid administration might help in intraoperative management and during early recovery (for instance the use of vasopressors to decrease further fluid administration), a weight gain threshold at POD 2 might serve as a useful point of reference on the surgical ward. In the authors' institution, care maps are used to facilitate and standardize care within the established enhanced recovery protocol. In patients who exceed the thresholds, subsequent preventive measures, such as fluid restriction, promotion of mobilization and diuretics, can be triggered.

This analysis has limitations as a result of its design and because it reflects a single institution's experience. Independent validation of these results is needed. The definition of thresholds was done not only by statistical means (ROC curve analysis) but also by clinical and pragmatic considerations, indicating some subjectivity. On that basis, the cut‐off values should be considered to provide quantitative guidance. Urine output, which might have impacted on postoperative weight gain, was not measured in the present study. The association between complications and fluid administration and weight gain cannot be seen as causal, as higher perioperative fluid administration might have reflected more difficult procedures or complications.

Independent validation of the proposed thresholds and prospective evaluation of treatments for unintended fluid overload are now needed.

## Disclosure

The authors declare no conflict of interest.

## Supporting information


**Table S1** Predictive values for perioperative fluid and weight gain at POD 2 thresholds
