# Supplementary material for: Thresholds for optimal fluid administration and weight gain after laparoscopic colorectal surgery
Source: BJS Open. 2019 Apr 2;3(4):532–8. doi: 10.1002/bjs5.50166 (PMC6677103; doi:10.1002/bjs5.50166)
Supplement: Supplementary file 1 — Table S1 Predictive values for perioperative fluid and weight gain at POD 2 thresholds [file BJS5-3-532-s001.docx]

**BJS5_50166**

**Thresholds for optimal fluid administration and weight gain after laparoscopic colorectal surgery**

**M. Hübner, B. Pache, J. Solà, C. Blanc, D. Hahnloser, N. Demartines and F. Grass**

**Table S1** Predictive values for perioperative fluid and weight gain at POD 2 thresholds

|  | **Sensitivity** | **Specificity** | **NPV** | **PPV** |
| --- | --- | --- | --- | --- |
| **Perioperative fluid** |  |  |  |  |
| Complications |  |  |  |  |
| Overall | 33 | 86 | 69 | 57 |
| Major | 43 | 81 | 94 | 16 |
| Respiratory | 57 | 80 | 98 | 11 |
| Ileus | 29 | 81 | 85 | 23 |
| **Weight gain** |  |  |  |  |
| Complications |  |  |  |  |
| Overall | 37 | 77 | 68 | 47 |
| Major | 33 | 72 | 94 | 8 |
| Respiratory | 67 | 73 | 98 | 9 |
| Ileus | 43 | 75 | 87 | 25 |

Predictive values associated with a) peri-operative fluids of > 3L (colon), resp. 4L (rectum) and b) weight gain of > 2.5kg at POD 2 for overall (Clavien I-V) complications (n=210, 36%), major (Clavien IIIb-V) complications (n=46, 8%), respiratory complications (n=23 patients, 4%) and postoperative ileus (n=98 patients, 17%).

NPV – negative predictive value, PPV – positive predictive value, POD – postoperative day
